# Applications of chemical imaging techniques in paleontology

**DOI:** 10.1093/nsr/nwy107

**Published:** 2018-10-10

**Authors:** Yanhong Pan, Liang Hu, Tao Zhao

**Affiliations:** 1 CAS Key Laboratory of Economic Stratigraphy and Palaeogeography, Nanjing Institute of Geology and Palaeontology and Center for Excellence in Life and Paleoenvironment, Chinese Academy of Sciences, Nanjing 210008, China; 2 University of Chinese Academy of Sciences, Beijing 100049, China; 3 State Key Laboratory of Palaeobiology and Stratigraphy, Nanjing Institute of Geology and Palaeontology and Center for Excellence in Life and Paleoenvironment, Chinese Academy of Sciences, Nanjing 210008, China

**Keywords:** trace elements, isotopic information, organic biomarkers, fossil materials, limits and obstacles

## Abstract

Chemical imaging techniques, based on a combination of microscopy and spectroscopy, are designed to analyse the composition and spatial distribution of heterogeneous chemical complexes within a sample. Over the last few decades, it has become an increasingly popular tool for characterizing trace elements, isotopic information and organic biomarkers (molecular biosignatures) found in fossils. Here, we introduce the analytical principle of each technique and the interpretation of the chemical signals, followed by a review of the main applications of these techniques in paleontology. We also demonstrate that each technique is associated with pros and cons, and the current limitations and obstacles associated with the use of each specific technique should be taken into account before being applied to fossil samples. Finally, we propose that, due to the rapid advances in the available technology and overall trends towards more multi-disciplinary studies in paleontology, chemical imaging techniques can be expected to have broader applications in paleontology in the near future.

## INTRODUCTION

‘A picture is worth a thousand words.’ Chemical imaging techniques combine spatial and chemical characterizations with images to provide a spatially resolved recording of elemental and molecular information. While conventional hyperspectral imaging only provides spatially resolved physical and morphological characteristics, chemical imaging creates a visual image with an integrated set of morphological, structural and chemical compositional information, even capturing the dynamics of some complex chemical processes with time-dependent spatial characterization in four dimensions (space and time) [1]. Over the last 10 years, chemical imaging has played an increasingly important role in chemistry, biology, medicine, pharmacy, food science, biotechnology, agriculture and industry [1]. The chemical characteristics of most materials are not homogeneous, so both ‘what’ and ‘where’ should be measured at millimeter to nanometer scales. This also holds true for fossils. No matter whether they are skeletal or soft-bodied, or preserved as molds (e.g. impressions, casts, mineralizations and compressions), or even isolated organs, fossils are most often chemically heterogeneous [2,3]. Most of the conventional analytical methods of chemistry have previously been applied in paleontology, such as coupled gas chromatography/mass spectrometry (GC/MS) [4–7] and coupled liquid chromatography/mass spectrometry (LC/MS) [8–10] and, as a result, the average chemical composition of a fossil sample can be obtained [3]. Without representative sampling and a proper understanding of the sample's heterogeneity, such results are compromised. Thus, in fossil studies, the advantage of chemical imaging over most conventional chemical analyses is the ability to identify and quantify chemical characters *in situ*. Identification and definition of the spatial distribution of the chemical compounds will enable paleontologists to further evaluate preservational states, characterize taphonomic alterations and identify the original biological information, which is critical to testing hypotheses about the phylogeny and evolution of organisms, and to understanding the true biological function and ecology of the fossilized structures [2,11–13]. Furthermore, such spatially resolved chemical patterns, extended to the interface between fossils and surrounding matrix and/or the interface between different parts of a fossil, can provide us with information regarding the fossilization and diagenetic processes acting upon them.

As fossils are valuable and irreplaceable resources, non-invasive analysis is essential where sampling is significantly restricted by the limited availability of specimens (many fossil species are known from only a single specimen). Even when sampling is possible, non-destructive analysis is generally preferred, which means the same sample or specimen can be preserved for further or repetitive analysis. There are several chemical imaging techniques that can be non-invasive, such as Fourier-transform infrared (FTIR) [14], X-ray fluorescence (XRF) [2] and Raman imaging [15], which usually scan the whole specimens or the whole sample without surface destruction, leaving the atoms or molecules in the sample intact. However, it is actually hard to define a surface-analysis technique as destructive or non-destructive. For example, energy-dispersive X-ray (EDX) and Raman analysis, theoretically, both do not remove the atoms or molecules on the surface, but they probably burn the sample surface due to the high-energy process involved. Moreover, preparing a smooth and polished sample surface for analysis can also in some ways be destructive. So, here, we refer to destructive chemical imaging techniques as those in which the atoms or molecules are sputtered away from the sample surface [16], such as laser-ablation methods [17] and secondary-ion mass spectroscopy (SIMS) [18,19]. Compared to the contraventional extraction analysis, in which the sample is either dissolved or degraded, these techniques are minimally destructive (only affecting the outermost surface of the sample, to a nanometer to millimeter depth) and will leave the sample intact for further analysis.

Due to these advantages, several chemical imaging techniques have been recently introduced into paleontology [3,20] and rapidly become important to the field. In general, each chemical imaging instrumentation has three components: a radiation source to illuminate the sample, a spectrally selective element, and a detector or a detector array to collect the images [1]. Here, we first describe the analytical principles of each technique and discuss the issues related to chemical signal formation and interpretation. Following, we will provide a review of the main applications of these techniques to fossil studies and the main advantages of each technique. Finally, we consider the intrinsic limits of each of these techniques and their potential shortcomings. The aim of this review is to underscore the great potential of label-free chemical imaging techniques (labeled imaging is beyond the scope of this review) in paleontology, compare various chemical imaging techniques that have already been applied to or have high potential to be utilized in paleontology in the near future and provide guidance in choosing a suitable method(s) for dealing with specific fossil samples and particular scientific questions. A side-by-side comparison of these chemical imaging techniques is provided in Table 1.

**Table 1:** Comparison of several chemical imaging techniques. Information is based on the usual values from fossil and rock studies.

Approaches	Source	Lateral resolution	Depth resolution	Chemical targets	Sample preparation	Vacuum	Sample damage induced
FTIR	IR light	2–10 μm	nanometers	C-H-O functional groups	polished and smooth surface; or transmitted thin sections for transmitted mold	no	non-destructive
Raman	laser beam	250 × 250 nm	microns	molecules showing Raman scattering phenomenon	flexible, rock chips or standard thin sections	no	non-destructive
XRF	X-ray beam	∼30 nm	nanometers	elements (reference-free)	flexible	no	non-destructive
XANES	X-ray beam	submicron	several microns	elements and the oxidation state	ultra-thin sections	no	non-destructive
EDS	electron beam	10 nm (SEM)1 nm (TEM)	microns (SEM) nanometers (TEM)	elements (reference required)	flexible for SEM mold; needs ultra-thin sections for TEM mold	high vacuum	non-destructive
EBSD	electron beam	20 nm5 nm transmission mold	surface	minerals	extremely high; the deformation of the crystallographic lattice at the surface should be minimized	high vacuum	non-destructive
LA-ICP-MS	laser beam	∼1 μm	several microns	elements, trace metals and isotopic information (reference required)	flexible	high vacuum	destructive
NanoSIMS	ions	50 nm	nanometers	elements, and isotopic information	polished, smooth surface	high vacuum	destructive
Tof-SIMS	ions	100 nm–5 μm	nanometers	elements and molecule fragments	smooth surface	high vacuum	destructive

## Non-destructive chemical imaging

### Fourier-transform infrared (FTIR) spectroscopy imaging

**Figure 1:**
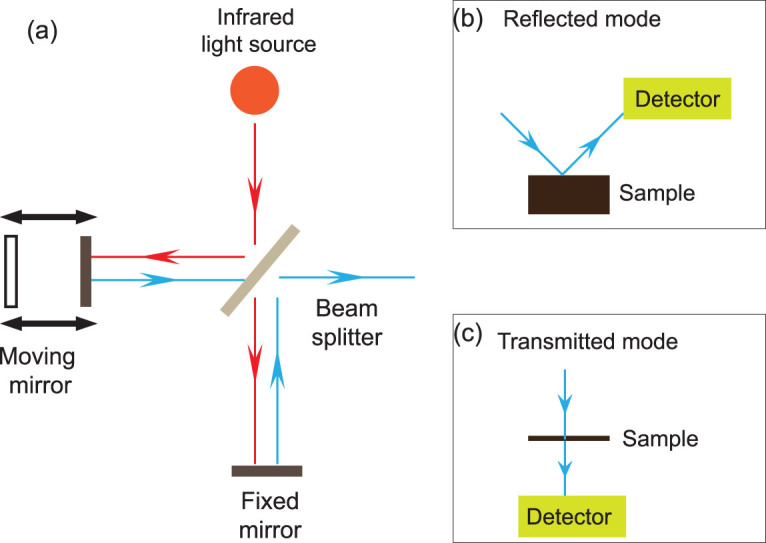
Schematic diagram of FTIR. (a) Schematic depiction of a FTIR system with an infrared source; (b) reflectance FTIR spectroscopy; (c) transmission FTIR spectroscopy.

**Figure 2:**
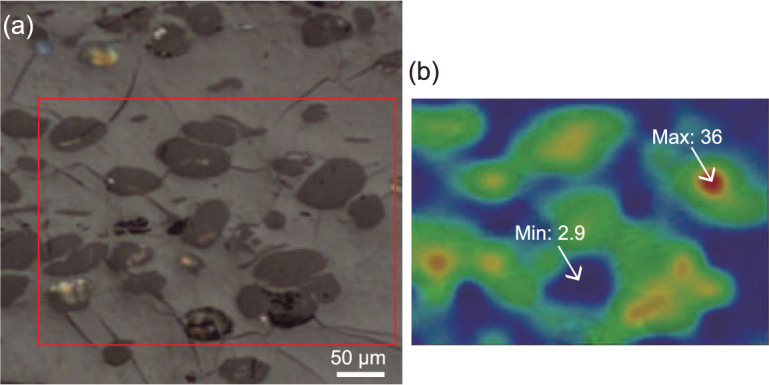
A FTIR imaging example of coal samples showing the chemical maps of resinite bodies in a vitrinite matrix. Adapted from [24]. (a) Micro-FTIR microscopic image—the mapped area is within the box; (b) mapping of CH_x_ groups (corresponding to the absorption region of 2800–3000 cm^−1^)—the maximum intensity of absorption is 36 and the minimum intensity of absorption is 2.9.

The FTIR technique is associated with transitions between quantized vibrational energy states [21]. The vibrations of chemical bonds (i.e. stretching, bending, twisting, rocking, wagging and out-of-plane deformation) are excited by the IR source (Fig. 1a), which then provides absorption information from the sample that is specific to a particular functional group (e.g. C-H, O-H, C = O, etc.) [22]. Two IR radiation molds are developed in FTIR techniques: transmission and reflectance (Fig. 1b and c). Although transmission mold provides a higher spatial resolution, reflectance mold can be directly applied on fossils. In the former mold, the sample must be thin enough to transmit light [23] while, in the latter, the thickness of the sample is not critical, but the topographical effects can be a problem [22]. The coupling of FTIR and visible-light microscopy makes the visualization and mapping of functional groups and abundances and molecular arrangements in samples across 2D regions [24], or even 3D cubes [25], possible. The infrared spectrum at each sampling point is measured and integrated so that spatial resolution is defined by the beam size of the IR [25]. Peak ranges corresponding to certain absorption bands or regions are then used to map the distributions of target functional groups [22,24] (Fig. 2). FTIR spectroscopy is based on the principle that different functional groups have different characteristic absorptions [26]. This can cause difficulties in the interpretation, especially when a large amount of chemical data is collected at the same time, forming a messy absorption spectrum [27].

FTIR spectroscopy coupled with imaging capabilities has been used to map the arrangements of molecules with H-C-O functional groups in fossil materials [28], since H-C-O functional groups are characterized by highly polar bonds and absorb infrared radiation with high efficiency. For a long time, FTIR spectroscopy has been used to detect the secondary structure of proteins in fossils [13,14,27,29,30] because it can provide the main parameters of the secondary structure of proteins through the absorption of the C = O (amide I) and N-H (amide II) groups (the corresponding IR bands are around 1655 and 1545 cm^−1^, respectively). For example, FTIR imaging of an Eocene reptile fossil skin (50 Ma) showed nearly identical distribution patterns of the absorption spectra at the amide I band and amide II band, which is also comparable to the extant control [14]. FTIR has also been applied to detect melanin preserved in fossils [10,31] and FTIR imaging of IR bands of melanin at 1580 cm^−1^ has faithfully replicated the feather patterns [31].

Although FTIR imaging is powerful, its disadvantages should also be considered. The main drawback of this technique is its limited spatial resolution (2–10 μm pertains to synchrotron light, around 50 μm to conventional light) [28,32]. Interpretation of spectral data depends on experienced spectroscopists. Unfortunately, interpretation errors occur quite often when IR spectroscopy is applied to fossils [27]. The ability to correctly interpret spectral data is a prerequisite for FTIR imaging. During surface analysis, only the first nanometers will be penetrated by IR photons in reflectance mode [26,27]. Thus, any contamination of the surface by exogenous organic matter would easily alter the quality of the results. Although IR spectroscopy shows the characteristics of functional groups of molecules, most molecules share the same functional groups. For example, all proteins have ‘C = O’ and ‘N-H’, so the spectra of amide I and amide II may indicate the remains of proteins, but not exactly certain specific proteins. This is also true of FTIR imaging.

**Figure 3:**
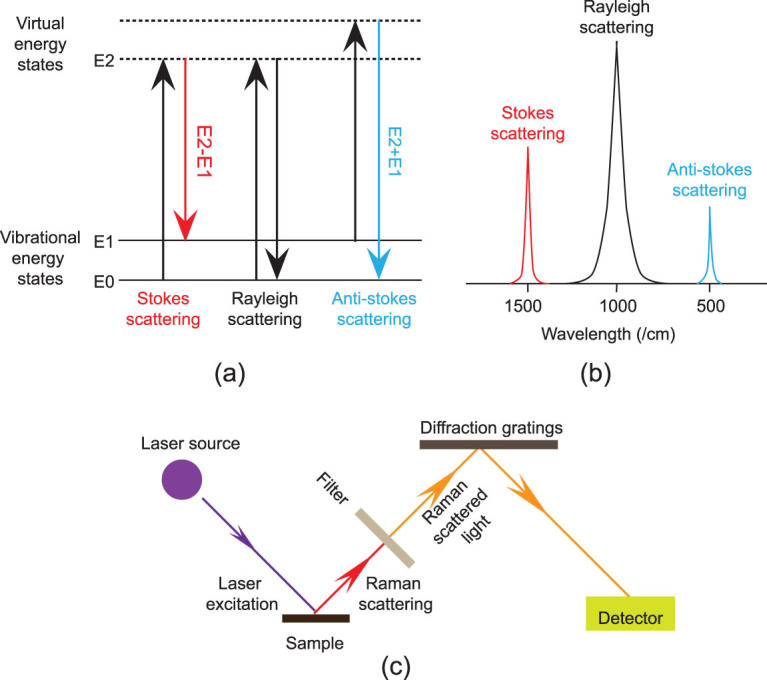
Schematic diagram of Raman spectroscopy. (a) Energy diagram of Rayleigh scattering (no exchange of energy), Stokes Raman scattering (molecule absorbs energy) and Anti-Stokes Raman scattering (molecule loses energy); (b) diagram of representative spectra of Rayleigh and Raman scattering; (c) the schematic depiction of a Raman spectroscopy system with a laser source.

### Raman spectroscopy imaging

Raman scattering is a scattering phenomenon defined by a two-photon event. When the photons are scattered from a molecule by an excitation, most of the scattered photons display Rayleigh scattering (the scattered photons have the same frequency as the incident photons), but a small fraction of them (the probability is 1:10^7^) show Raman scattering (the scattered photons have a frequency different from the incident photons). There are two types of Raman scattering: Stokes scattering (the molecule absorbs energy) and anti-Stokes scattering (the molecule loses energy) [33,34] (Fig. 3a and b). In Raman spectroscopy, unlike IR spectroscopy, the sample is irradiated with mono-wavelength laser light and the scattered light is then collected with an arrangement of optics and a detector (Fig. 3c). The frequency of light scattered from a molecule is dependent on the structural characteristics of the chemical bands, so each peak corresponds to a given Raman shift (from the incident radiation energy) related to a specific molecular vibration and represents a particular chemical constituent of the sample [35].

Raman spectroscopy has been developed into a powerful imaging technique [36,37], due to its high potential to allow quantitative evaluation of carbon chemistry, the non-invasive nature of the analysis leaving the chemistry and morphology of the sample intact, and the minimal specimen preparation required (applicable to rock chips and standard uncovered geological thin sections) [32]. The achievable spatial resolution is down to 20–50 nm when coupled with confocal microscopy, near-field optical techniques or super resolution imaging techniques (e.g. Scanning-Field OM) [26]. Raman spectroscopy data are acquired via laser excitation of the chemical bonds within the samples; when it is coupled with confocal imaging, 3D chemical and structural maps can be achieved [32]. Raman spectroscopy imaging is favored for investigating the preservation of organic matter within its inorganic framework in geological samples [38,39] and for revealing the heterogeneity and potential biotic origin of the organic matter based on biological structure and chemical composition in fossils [15,37,40,41]. For example, Raman imaging on Proterozoic acritarchs and algae provided information about their morphology, cellular anatomy, taphonomy, carbonaceous composition and geochemical maturity [32,37].

The following are the challenges and limitations of Raman spectroscopy imaging. The Raman signal is usually weak [34], so efficient Raman imaging requires powerful irradiation over a relatively long time. Laser is used as the excitation source; the heat generated by the laser exposure may damage sensitive biomolecules such as proteins and enzymes. Furthermore, thermal emissions due to sample heating may cause unwanted background effects that can be superimposed on the Raman spectrum [42]. Imaging an auto-fluorescenced sample is a challenge due to the strong fluorescence of the backgrounds, which can completely overwhelm the Raman signal [35]. The technique detects the signals from the subsurface rather than the surface, so the resultant information is not from the top molecular layer of the sample.

### XRF imaging

**Figure 4:**
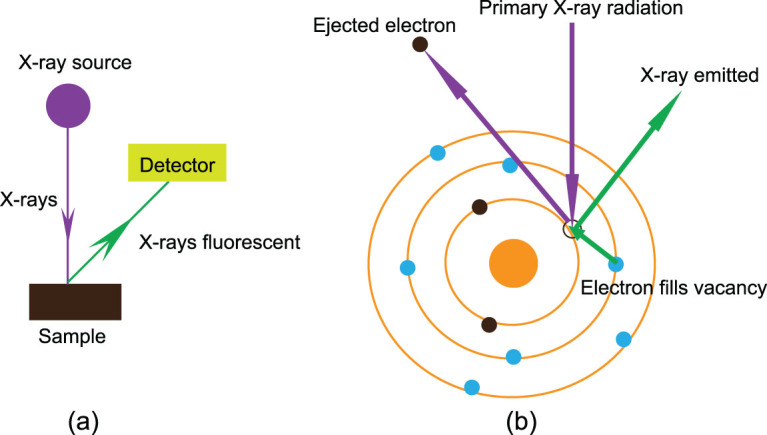
Schematic diagram of XRF spectroscopy. (a) The schematic depiction of an XRF spectroscopy system with an X-ray source; (b) simplified diagram of the principle of X-ray-excited fluorescence.

When a sample is excited by a primary X-ray source, some electrons are knocked out of their orbit leaving behind vacancies, which are immediately filled with the electrons from the next orbit. In this way, the fluorescent (or secondary) X-ray will be emitted, which can be measured by XRF analysers (Fig. 4). XRF spectroscopy has long been known to be a sensitive, quantitative tool for studying the elemental composition of materials [43] because each specific element in a sample produces a unique set of characteristic fluorescent X-rays. Imaging analysis of materials with XRF at the microscopic and sub-microscopic levels has grown significantly, especially in relation to the application of synchrotron radiation (SR). This technique is referred to as synchrotron rapid scanning X-ray fluorescence (SRS-XRF) [2,14,44].

**Figure 5:**
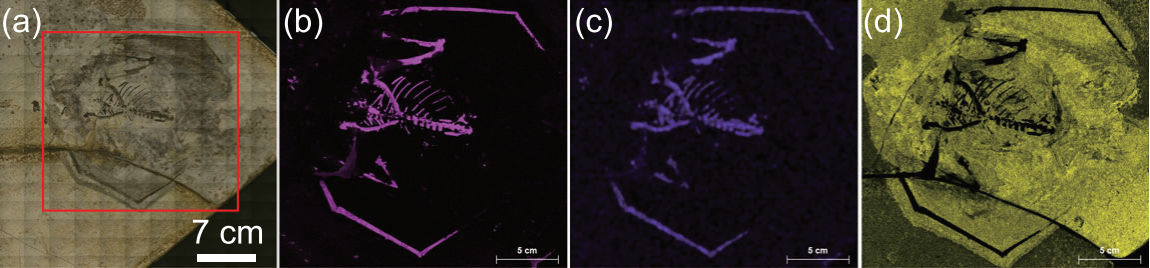
Macro-XRF imaging example of a fossil specimen (IVPP V12705) showing the elemental distribution maps. (a) The mapped area is within the box; (b) calcium (Ca) distribution map; (c) phosphorus (P) distribution map; (d) potassium (K) distribution map.

XRF is a reference-free quantitative technique, which forms the base of the most accurate chemical imaging tools [45]. Currently, XRF analysis is developed as an accurate reference method or a primary method for relative methods that require comparison with certified reference materials [46]. SRS-XPF is now routinely performed with a spatial resolution below the 1-μm scale [45,47], while the limits of detection may decrease to approximately 1 ppm for heavier elements [2,44]. The scan time now can be reduced to ∼ 30 seconds/cm^2^ at 100-um resolution at the Stanford Synchrotron Radiation Lightsource (SSRL) [44]. Another major advantage is that X-ray techniques do not require the high vacuum conditions necessary for many other analytical techniques, thus allowing the measurement of samples in their natural (e.g. wet) conditions.

SRS-XRF imaging of fossil materials has been carried out at SSRL [2,14,20,48] and at the Diamond Light Source (DLS, Oxford, UK) [13]. SRS-XRF imaging was first applied on an *Archaeopteryx* specimen, revealing the chemical composition of the feathers and bones [44]. SRS-XRF imaging has been successfully used to detect elements and trace metals as biomarkers for eumelanin pigments in various fossil feathers [2,20,48]. It has also been applied to other fossil soft tissues, including supposed soft tissues with typical proteoglycan aggrecan and collagen preserved in a *Confuciusornis* specimen (Early Cretaceous bird, Yixian Formation) [13] and to a ∼50-Ma reptile skin from the Green River Formation (USA) with possible keratin remains [14].

Besides SRS-XRF, macro-analytical XRF scanning (MA-XRF) is a variant of XRF imaging that allows the distribution of elements to be visualized at the macroscopic level. This is achieved using a focused X-ray beam with a millimeter or larger size to scan the entire surface of the macroscopic sample and analysing the emitted fluorescent radiation [49] (Fig. 5). Compared to SRS-XRF, MA-XRF has a much lower spatial resolution but the equipment is portable and can be taken to museums or other locations for on-site analysis. A similar scanning method has been widely applied to fossil specimens, but instead of a focused X-ray beam, the high flux of a laser is used to induce the fluorescence (Laser-stimulated Fluorescence, LSF) in the different components comprising the fossils [50]. Through this technique, many specimens have been shown to preserve not simply impressions, but also chemical residues perhaps representing both soft and hard tissues [51–53]. However, no chemical information is acquired through LSF.

The challenges and limitations of XRF imaging are as follows. As a reference-free analysis, it requires a prior knowledge of the fundamental parameters including photoionization cross-sections, X-ray, Auger and Coster-Kronig transition rates, fluorescence yields and vacancy transfer probabilities [45]. Therefore, extensive experimental work, calculations and critical complications are essential to obtain these fundamental parameters [45]. Due to the penetrative nature of X-rays, elements present at and below the surface contribute to the obtained elemental distribution images. There are a limited number of light sources capable of providing SRS-XRF and there is a competitive application process for access to the equipped light sources. For MA-XRF, due to the difficulty in focusing X-rays, the current spatial resolution limits the range of potential applications.

### X-ray absorption near edge structure (XANES) imaging

**Figure 6:**
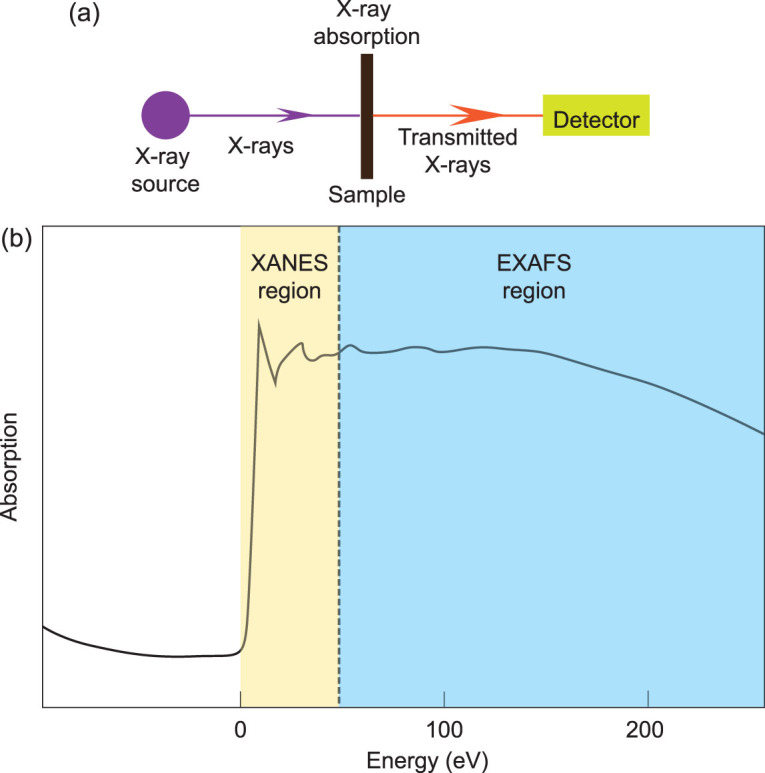
Schematic diagram of XANES spectroscopy and an X-ray absorption spectrum. (a) Schematic depiction of a XANES spectroscopy system with an X-ray source; (b) schematic illustration of an X-ray absorption spectrum including XANES and EXAFS regions.

In XAS, the fine structure of the X-ray absorption edge depends on the local chemical environment and the state of the excited atom (Fig. 6), such as its oxidation state, site ligation and coordination [45]. The XAS spectrum is mainly composed of two regions: the XANES region and the EXAFS region (Fig. 6) [54]. The fine structure of the XANES region is sensitive to the electronic structure of the probed absorber atom, especially to oxidation state and coordination number [45]. Thus, maps of XANES region-sensitive molecules would be provided by XANES imaging.

XANES has mainly been applied to determine the oxidation states of elements in fossil studies, such as in discriminating the sulfur oxidation states in the fossil feather of *Archaeopteryx* [2]. However, in this pioneer work on fossils, XANES was not used as an imaging tool, but rather combined with XRF imaging. For instance, XRF imaging was first applied and then XANES spectra were collected from specific regions of interest within the XRF map and using XANES spectroscopy at the Kα edge of the studied elements (e.g. S, Cu) to determine their oxidation states [2,13,14,20,44,55]. XANES mapping was first applied to fossils to characterize the molecular signature of the chitin–protein complex in the cuticle of a Paleozoic arthropod [12]. The fossil cuticle was analysed by employing functional group-sensitive imaging and submicron spatial resolution XANES spectroscopy, using a synchrotron-based scanning transmission X-ray microscope (STXM) [56].

Challenges and limitations of XANES imaging are as follows. STXM-XANES imaging is done by the transmission mode, requiring the sample to be prepared into ultra-thin sections (100–150 nm) for the X-rays to transmit. If the sample is thick, the collected data will represent the average information from the analysed sample volume. The other drawbacks are the slow rate of data collection, as well as limited access to SR facilities and experimental time constraints similar to XRF imaging. Fluorescence XANES imaging is another mode that has yet to be applied on fossil material. Compared to the transmission mode, it does not have thickness and/or related low analytical concentration constraints. It can be applied to the surface layers of thick, non-transparent samples [57], especially with the development of fast XRF detectors that facilitate fast scanning, allowing rapid collection of images [58].

### EDX imaging

The principle of energy-dispersive X-ray spectrometry (EDX, also used as EDS) is similar to XRF [1,59]. When combined with an electron microscope, the sample is excited by a focused electron beam and some electrons are knocked out of their orbit, leaving behind vacancies, which are immediately filled with the electrons from the next orbit. In this way, the characteristic X-ray spectra are produced, which can be detected and investigated [59]. After being scanned by the beam, the investigated area will display the intensity of a selected characteristic X-ray line corresponding to a certain element, which makes it possible to produce element distribution maps [60,61].

**Figure 7:**
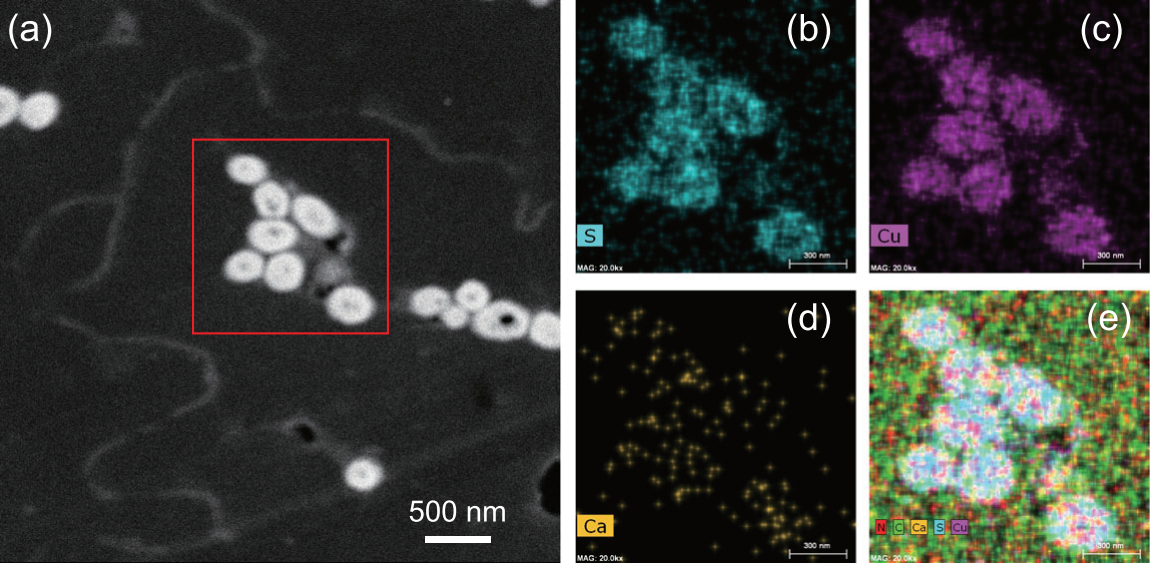
A STEM/EDX imaging example of the ultrastructure of a feather sample. Adapted from [71]. (a) TEM image of a feather from *G. gallus*; box shows area selected for STEM/EDX imaging; (b) map of sulfur (S); (c) map of copper (Cu); (d) map of calcium (Ca); (e) overlapped maps of S, Cu, Ca, carbon (C) and nitrogen (N).

EDX spectroscopy, especially when combined with scanning electron microscopy (SEM/EDX), is one of the best-known and most widely applied elemental imaging analytical techniques used on fossils [62–67]. SEM imaging with secondary electron (SE) or backscattered electron (BSE) excitation is accompanied by X-ray analysis and is considered a relatively rapid, inexpensive and basically non-destructive approach [61,68]. It is often used to survey surface analytical problems before proceeding to techniques that are more surface-sensitive and more specialized [60]. Besides SEM, EDX has also matured into TEM and STEM, both used as chemical imaging tools. The most obvious advantage of TEM is that it captures the greatest spatial resolution, making it possible to image at the atomic level. The steady improvement of TEM and STEM with EDX has allowed scientists to perform structural determinations at atomic resolution [69,70]. For TEM, preparation of ultra-thin sections (<100 nm) is essential, and this is a major challenge for fossil materials. The conventional method to cut ultra-thin sections uses a microtome and a diamond knife—a technique that requires experience [71]. Recently, the combination of focused ion beams with SEM (FIB-SEM) has provided a new method of sample preparation for TEM observation [72] that can also be applied to TEM and STEM/EDX imaging [73–75] (Fig. 7). The advantage of TEM and STEM/EDX is the high spatial resolution (nano-scale) and the short time requirement, especially in the ChemiSTEM mold [32,71].

Although EDX imaging is one of the most commonly used chemical imaging techniques in paleontology, it should be noted that there are limitations of EDX imaging. The light elements, especially the first three elements of the periodic table, are not detectable by EDX [59]. Furthermore, owing to the low spectral resolution, many elements have overlapping peaks in the EDX spectrum [59]. Compared to the SIMS, the accuracy and sensitivity of EDX are much lower, commonly obtaining a precision of about ± 2% [60]. For more detail on the challenges and limitations of EDX imaging, see [60,61].

### Electron backscattered diffraction (EBSD) imaging

**Figure 8:**
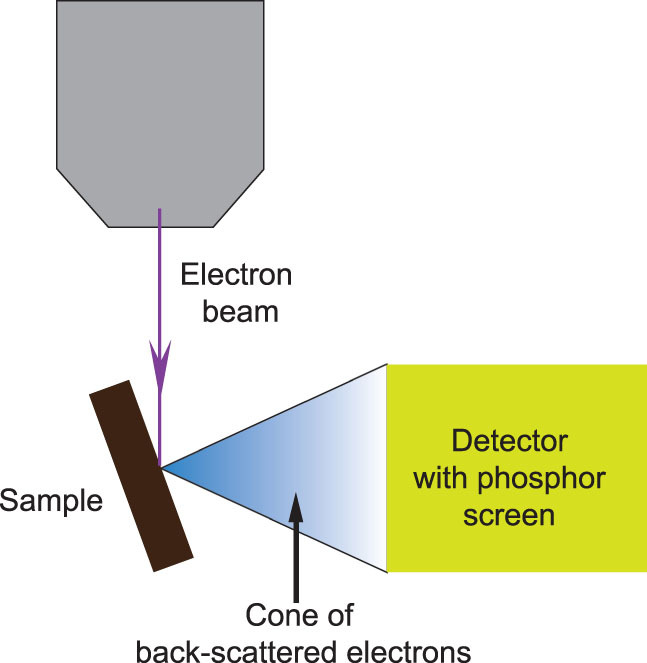
Schematic illustration of an EBSD system.

EBSD is a high-energy electron-beam technique based on the elastic scattering of a focused electron beam on a strictly polished surface, which is used to characterize the crystalline structure of heterogeneous materials at the millimeter to nanometer scale when combined with SEM [60,76]. This technique relies on a finely focused electron-beam incident onto a stage-tilted sample oriented at 70° to the normal of the incident electron beam. An EBSD pattern emanates from the point of interaction to the electron beam and is imaged on a phosphor screen (Fig. 8) [60]. This diffraction pattern (also called the Kikuchi pattern) is composed of lines and bands of contrasts of electron intensity, which is uniquely defined by lattice parameters of the crystal under the beam and by the orientation of the crystal in space. In an EBSD detector system, a sensitive charge coupled device (CCD) camera is used for viewing and digitizing the diffraction patterns on the phosphor screen, after which the collected raw data are analysed. By selecting expected crystal phases from a phase database until the best fit for all possible identities and orientations of crystals is found, the Kikuchi pattern is then considered indexed, and the orientation and phase of the crystal at the interaction point on the sample are reported. The crystallographic phase and orientation of each measuring point of the scanned area would be recorded and integrated into phase and orientation images.

**Figure 9:**
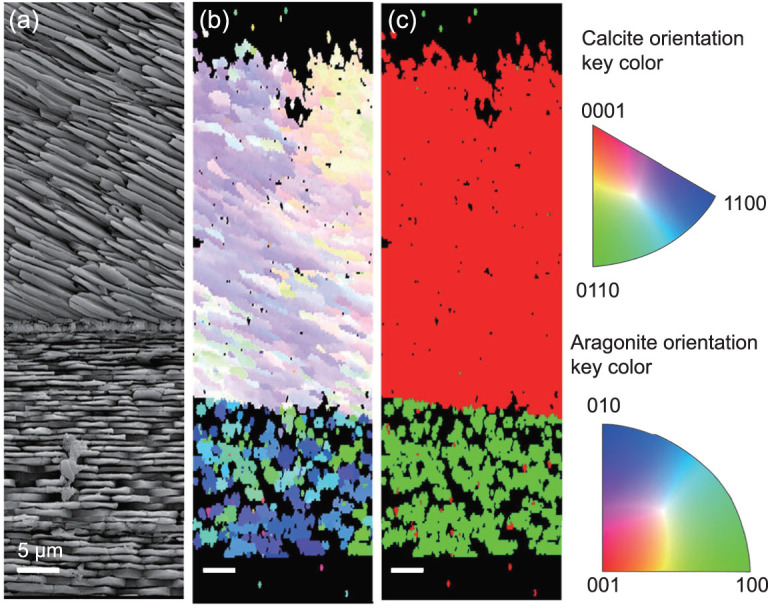
An EBSD imaging example of the cross-section of a mussel shell. Adapted from [113]. (a) SEM images of the shell; (b) the crystallographic orientation map of the calcite (top) and aragonite (bottom) according to color key; (c) phase map where calcite is in red and aragonite in green. Scale bar = 5 µm.

EBSD has been utilized in the biomineral research of both modern and fossil organisms to identify mineral polymorph and crystallographic orientation *in situ* (Fig. 9). For a discussion of the applications to biological specimens, see [77,78]. In fossil studies, this technique helps to identify original and secondary minerals even when the secondary mineral alludes to having the same mineral composition as the original. For example, it can be used to discriminate diagenetic mineralization that could distort paleoclimate calculations [77,79]. Additionally, EBSD can also be employed to distinguish biologically induced and biologically controlled calcareous biomineralization in fossil organisms [80], to evaluate the degree of diagenetic alteration in modern and fossil brachiopod shell calcite [81], to study the crystallographic and ultrastructural features of dinosaur egg shells [82] and to investigate the microstructure, crystallography and diagenetic alteration in fossil ostrich egg shells [83].

In fossil studies, the images of crystallographic orientations of the biomineral microstructures acquired by EBSD is quite important, because they provide information about the relationships between diverse elements, the processes of growth and the architectural responses that lead to ecophenotypical variation [82,84–87], as well as the effects of diagenesis [79,87,88].

By scanning the beam over the sample at steadily increasing speeds, EBSD creates an orientation map. Currently, this is the fastest and most reliable way to acquire data regarding the structure and orientation of minerals in a solid crystalline specimen. However, the challenges and limitations are also obvious. The spatial resolution of the traditional EBSD combined SEM is limited to 25–100 nm [78]. A new approach referred to as transmission EBSD or SEM transmission Kikuchi Diffraction (TKD) provides a spatial resolution of better than 10 nm. But the TKD technique requires that the sample be thin enough to be electron transparent. Specimen preparation for EBSD is very critical; diffracted electrons escape from the top surface layer, which is only a few tens of nanometers of the specimen surface.

## Destructive chemical imaging

### Laser ablation inductively coupled plasma mass spectrometry (LA-ICP-MS) imaging

**Figure 10:**
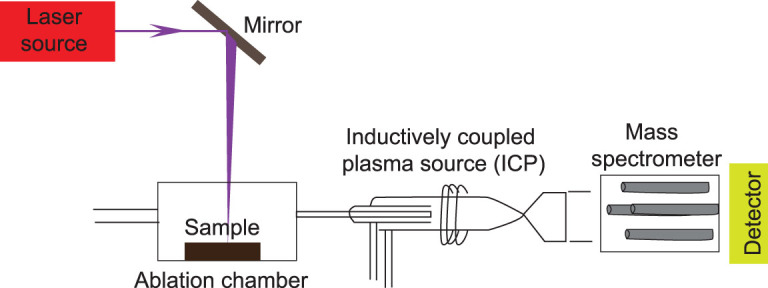
Schematic illustration of a LA-ICP-MS system.

LA-ICP-MS imaging is based on the use of a pulsed and focused laser beam directed on the surface of the sample. The sample stage is usually moved at a defined speed, passing through a series of successive laser pulses, after which the ablated material is swept to the ICP by a carrier gas, where positive ions are produced in the plasma, which is then transferred into the vacuum of the mass analyser (Fig. 10).

The advantage of LA-ICP-MS is its high speed, multi-element capacity, ultra-trace sensitivity (some elements can be measured down to the part-per-quadrillion (ppq) level and most can be detected at the part-per-trillion (ppt) level) [89], relatively high accuracy and precision of the analytical data, and potential for isotopic information. In addition, because LA-ICP-MS is without charging effects, it is at a low risk of foreign contamination and has few matrix effects, simplifying quantification of analytical data compared to SIMS, which requires a charged surface [90]. Also, compared to synchrotron-based XRF, LA-ICP-MS is significantly less expensive and allows isotope ratios and metal concentrations to be measured without restrictions at lower concentration levels. LA-ICP-MS is becoming the method of choice as a sensitive inorganic mass spectrometric technique for the imaging of metals and non-metals at trace-concentration levels in material, environmental, geological, biological and clinical research [17,91,92].

LA-ICP-MS was used to reconstruct the diet and migration pattern of prehistoric brown bears by analysing the distribution of minor and trace elements and matrix components in the dentine of a fossilized tooth [91]. However, LA-ICP-MS has not been commonly used in fossil studies because of the obvious challenges and limitations.

Differing from any techniques described above, it is a destructive analysis. Laser ablation will destroy the surface of the sample (even several microns deep). It has relatively low spatial resolution, at most down to 1 micron. LA-ICP-MS is considered a panoramic elemental analytical method, but elements such as F, O, H, N, S, Si, Se, As and Ca remain difficult to measure.

**Figure 11:**
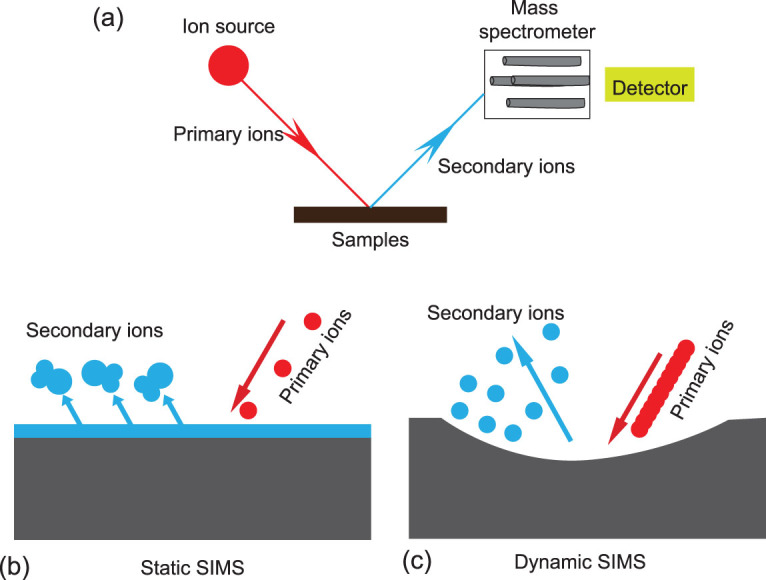
Schematic illustration of a SIMS system (a). Schematic diagram of static SIMS (b); schematic diagram of dynamic SIMS (c).

### NanoSIMS imaging

SIMS is a technique capable of providing information about the molecular, elemental and isotopic composition of a sample *in situ* from a few micrometers down to the submicron scale with great sensitivity (i.e. even when only present at the parts-per-billion level) [32]. The surface of a sample is typically sputtered with an energetic primary ion beam, leading to collision cascades that break the chemical bonds of the matter, generating the ejection of the secondary ions from the sample surface (Fig. 11). The emitted secondary ions are then collected and analysed using a mass spectrometer (Fig. 11; for more details, see Ireland, 2013 [93]). The intensities of the secondary ions that are characteristics of specific components create an image of that component's distribution on the surface of the sample. Aside from elemental detection, the SIMS technique is capable of analysing isotopic and organic molecular compositions [94]. Depending on the ion dose applied to the sample, there are two distinct modes of operation, corresponding to two different types of instruments defined as dynamic (D-) and static (S-) SIMS (Fig. 11). NanoSIMS is a classic representative of D-SIMS, while time of flight-SIMS (Tof-SIMS) is a form of S-SIMS.

The most notable advantages of NanoSIMS are its high spatial resolution, capable of element (ion) imaging with a lateral resolution down to ∼50 nm (see Kilburn and Wacey, 2015 [95] for details) [19,95], and its high transmission at high mass resolution (60% at M/ΔM = 4000), which can differentiate isobars of the same nominal mass (i.e. ^12^C^15^N^−^, 26.9996 amu; and ^13^C^14^N^−^, 27.0059 amu can be separated) [96]. NanoSIMS imaging has been applied to the studies of Proterozoic microfossils [19,97,98]; for example, it hs been used to reveal the isotopic and chemical heterogeneities within morphological features, indicating that the Proterozoic cyanobacterial cell walls have been preserved nearly intact [98].

The challenges and limitations for NanoSIMS are as follows. NanoSIMS can also give accurate isotopic measurements for samples <5 um, although it is less precise (generally >1‰) than the large-radius secondary-ion mass spectrometer. NanoSIMS is limited to elemental analysis and is not suitable for organic molecular analysis.

### Tof-SIMS imaging

A generic scheme of Tof-SIMS is similar to all other SIMS experiments. As has been explained above, Tof-SIMS offers an alternative to D-SIMS, in which the analysis is completed before the incoming ions have significantly damaged the sample surface [3,99]. Tof-SIMS has been used for approximately two decades for surface analysis [99], but is limited to the inorganic or polymer surface analysis. The low sensitivity and large amount of molecular fragmentation associated with this technique are major problems when it comes to analysing organic materials [100]. However, over recent decades, the use of cluster primary ion sources has greatly improved the ability of this technique to obtain information from organic molecules [18,101–103]. The cluster of primary ion sources reduces the fragmentation considerably, thereby enabling the analysis of molecular species of up to ∼2000 Da, with a lateral resolution of a few hundred nanometers [100,101]. Thus, Tof-SIMS is a mass spectrometry technique capable of producing fine spatial resolution for organic components on a scanned surface [101,104,105]. This allows an unlabeled molecule that produces secondary ions with distinctive m/z to be detected and imaged. Compared to other imaging techniques, Tof-SIMS is capable of analysing relatively large ions with masses as high as several thousand atomic mass units (optimum mass range 0–2000 amu) and has the potential to achieve sub-micrometer lateral resolution, which may allow the use of biomarkers at the microscopic level [3].

**Figure 12:**
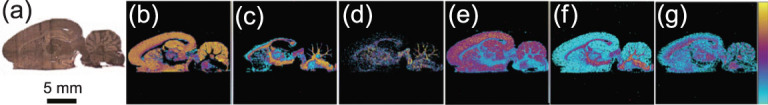
A Tof-SIMS imaging example of a rat-brain section. Adapted from [106]. (a) Optical image of the rat-brain section; (b)–(d) positive ion images; (b) m/z 184.1; (c) m/z 385.4; (d) m/z 796.8; (e)–(g) negative ion images; (e) m/z 283.2; (f) m/z 429.3; (g) m/z 888.9.

Cluster Tof-SIMS imaging has been used to investigate various aspects of lipids with regard to biology and medicine [101,106] (Fig. 12). This technique has also been applied in the study of cultural artifacts, such as the various materials used for painting [102,103]. Tof-SIMS imaging has also been used to detect the protein composition of the skin of a human mummy [18].

The first application of Tof-SIMS in paleontology was the study of beta keratin preserved in the short fibers of the Late Cretaceous theropod dinosaur *Shuvuuia deserti* [107]. Since then, Tof-SIMS has been used to detect the protein breakdown bi-products preserved in a variety of fossil specimens, including claw sheath from a Late Cretaceous bird [108], collagen and blood in dinosaur bones [109], Early Cretaceous bird cartilage [13], melanin in fish eye [110], feathers, squid ink, amphibian skin, etc. [11,111,112].

Despite rapid improvements in the use of this technique and its wide variety of applications, the use of Tof-SIMS on organic compounds in fossil samples remains a challenge. Unfortunately, molecular ions of large molecules cannot be detected using Tof-SIMS, which generally produces both elements and small molecular fragments. When the mass resolution is better than 0.05 amu, the analyst will get a Tof-SIMS spectrum representing the complex assemblage of detectable compounds, because no upstream separation is applied [3]. Thus, interpretation of Tof-SIMS spectra is to some extent based on probabilistic reasoning [3]. To take large molecular proteins as an example, this technique generally produces fragments of proteins as small as individual amino acids or amino acid components [3,9]. However, the use of Tof-SIMS is less problematic when the target protein has chemical signature ions. For instance, it has been used to recognize the keratin and collagen from human mummy skin, in which the localization of characteristic amino acid fragments in the sample was imaged by Tof-SIMS [18]. For example, the presence of hydroxyproline, an amino acid that is only found in collagen but not in keratin and leucine/isoleucine fragments characterizing keratin have been documented [18].

However, there are other challenges and limitations of Tof-SIMS. A reasonably flat surface is preferred due to topographic effects and the structures to be analysed should be fully exposed, since only the very surface of the sample is analysed [3]. Tof-SIMS breaks large molecules apart so that any modifications to molecules caused by diagenesis cannot be observed using this technique [9]. Since only the very surface of the sample is analysed, it is difficult to differentiate surface contamination from an endogenic signal [9]. For more detailed instructions as well as limitations, see [3].

## CONCLUSIONS

The high-resolution chemical imaging techniques currently applicable to paleontology have been reviewed here. The techniques have been classified as either non-destructive analyses (e.g. FTIR, Raman spectroscopy, X-ray spectroscopy, SEM/TEM EDS and EBSD) or as destructive analyses (e.g. LA-ICP-MS, NanoSIMS and Tof-SIMS).

Each technique has its own pros and cons, as well as specific limitations in their applicability to various types of fossil samples. For example, the X-ray source equipped XRF and XANES have a relatively high resolution and sensitivity, but access to the X-ray source is limited; EDS combined with TEM provides the greatest spatial resolution in elemental mapping, but sample preparation is an obstacle in most fossil materials; although EBSD uniquely provides maps of mineral characters, the analysed surface is extremely restricted; and NanoSIMS provides high spatial resolution and even isotopic data, but it is not suitable for organic detecting and has limited abilities for quantitative analysis. While some chemical imaging methods are highly dependent on vacuum conditions (e.g. ToF-SIMS and NanoSIMS), methods such as XRF and LA-ICP-MS have a considerable advantage in this respect. In general, four factors are of importance with regard to various chemical imaging techniques: spatial resolution, spectral resolution, field of view and magnification. Thus, choosing a proper method with the required spatial resolution and spectral resolution for a proper chemical target is critical. Finally, it is comparatively rare that a single experimental approach is able to satisfactorily answer a diverse set of scientific questions and multiple techniques are often required in the study of fossil materials.

There are growing techniques on chemical imaging methods on material sciences and biology. For example, imaging combined with scan probe microscopy (e.g. scanning tunneling microscopy and atomic force microscopy) and imaging based on ionization, such as matrix-assisted laser desorption ionization (MALDI) and desorption electrospray ionization (DESI) imaging, which will also have great potential to be used in paleontology. Besides testing and application of these untested analytical techniques, new attempts at combining performance of various imaging techniques on the same sample are now considered in paleontology, providing elemental, chemical, molecular and morphological information at nanometer to microtome scales. The techniques applied on fossils are mainly qualitative; good quantitative imaging has not yet been achieved. With rapid development, quantitative imaging will not be far beyond.

Thanks to the recent rapid technological developments, we are optimistic that new generations of improved chemical imaging techniques and innovative methods will appear and will continue to play an ever-increasing role in paleontological studies. Furthermore, it is an ongoing trend that paleontology is becoming increasingly more integrated with other disciplines of geology and biology, and chemical imaging techniques are expected to have a broader application in multi-disciplinary studies of fossil samples to reveal tremendous amounts of new information about the history of life and its environmental background in the near future. Honestly, it is impossible for one person or group to have in-depth knowledge of or master all the techniques. Thus, the expansion of this field in paleontology will greatly encourage multi-disciplinary cooperation.
